# Serum Sphingomyelin and Osteoarthritis Risk: Evidence from Mendelian Randomization, Genetic Colocalization, and a Rat Model

**DOI:** 10.3390/biomedicines14102335

**Published:** 2026-10-14

**Authors:** Yulin Jin, Abudukeremu Dawuti, Zeyang Zhang, Hui Zhao, Chao Zhang

**Affiliations:** 1 The First Clinical Medical College, Lanzhou University, Lanzhou 730000, China; jyl1842710521@163.com (Y.J.);; 2 Department of Orthopaedics, the First Hospital of Lanzhou University, Lanzhou 730000, China

**Keywords:** osteoarthritis, sphingomyelin, Mendelian randomization, genetic colocalization, destabilization of the medial meniscus, lipid metabolism

## Abstract

**Background**: Sphingomyelin (SM) is a major constituent of circulating lipoproteins and cell membranes, yet its role in osteoarthritis (OA) remains unclear. This study integrated Mendelian randomization (MR), genetic colocalization, and animal experiments to investigate the association of serum SM with OA risk. **Methods**: Using GWAS summary statistics for serum SM and four OA phenotypes, we conducted two-sample MR with inverse-variance weighting as the primary method, complemented by weighted median, MR-Egger, and weighted mode, as well as sensitivity and reverse MR analyses, and applied colocalization to identify shared genetic signals. The animal experiment comprised four groups of male Sprague–Dawley rats defined by surgical procedure (DMM or sham) and treatment (SM or vehicle), with six rats per group. Following the intervention, histology and micro-CT assessed structural joint alterations, while RT-qPCR and Western blotting examined molecular changes. **Results**: Genetically predicted serum SM was positively associated with overall OA (OR per SD increase, 1.039; 95% CI, 1.020–1.059), hip OA (OR, 1.147; 95% CI, 1.118–1.177), knee OA (OR, 1.035; 95% CI, 1.011–1.060), and other OA (OR, 1.029; 95% CI, 1.009–1.050). The associations were generally consistent across sensitivity analyses, with no evidence of horizontal pleiotropy or undue influence from individual variants, while reverse MR yielded no consistent evidence that genetic liability to OA affected serum SM. Twenty-one locus–outcome pairs showed strong evidence of shared genetic signals, with the strongest colocalization for knee OA at Chr6:43.26–44.26 Mb (PP.H4 = 0.999). Compared with vehicle, SM treatment exacerbated cartilage degeneration and subchondral bone microarchitecture in DMM rats. Molecularly, this phenotype was characterized by upregulated cartilage expression of *Mmp13*, *IL-1β*, *Smpdl3a*, and *Cd36*; higher MMP13 and cleaved caspase-3 protein levels; and increases in both total and phosphorylated p38. But the phosphorylated-to-total p38 ratio did not differ significantly between groups. **Conclusions**: Higher genetically predicted serum SM was associated with greater OA risk, while its exogenous administration exacerbated DMM-induced joint degeneration in rats, collectively positioning elevated SM as a candidate metabolic factor in OA pathogenesis.

## 1. Introduction

Osteoarthritis (OA) is a leading cause of pain, disability, and reduced quality of life among middle-aged and older adults, while effective disease-modifying treatments remain unavailable [1,2,3,4,5]. Accumulating evidence indicates that OA involves the entire joint and is shaped not only by mechanical stress but also by chronic low-grade inflammation and metabolic dysregulation [6,7,8]. In addition to articular cartilage, the synovium, subchondral bone, menisci, and periarticular tissues are involved in OA-related pathological changes. Excessive or abnormal mechanical stimuli may promote catabolic responses in cartilage, whereas inflammatory and metabolic abnormalities may further impair the ability of joint tissues to adapt to mechanical stress [9,10]. The associations of OA with obesity, diabetes, and metabolic syndrome further support a systemic metabolic contribution to disease development [11,12,13]. Within this framework, circulating metabolites may act as bioactive mediators of joint homeostasis, consistent with the altered lipid profiles identified in the serum, synovial fluid, and joint tissues of patients with OA, particularly involving sphingolipids and glycerophospholipids [11,14,15,16,17].

Beyond its structural role in cell membranes and circulating lipoproteins, sphingomyelin (SM) is metabolically linked to bioactive sphingolipids, including ceramide and sphingosine-1-phosphate [18,19]. SM turnover within the sphingolipid network connects membrane organization with signaling pathways implicated in oxidative stress, apoptosis, and autophagy, supporting its potential relevance to OA [18,19,20]. However, available evidence comes largely from cross-sectional observations, lipidomic association analyses, and in vitro experiments, making it difficult to adequately exclude confounding and reverse causation [15,16,17,20]. Whether elevated serum SM is an upstream risk factor for OA or a metabolic alteration accompanying the disease remains to be determined.

By using germline genetic variants as instruments, Mendelian randomization (MR) can help assess the potential causal contribution of circulating SM to OA while addressing some limitations of conventional observational studies [21,22,23]. On this basis, we hypothesized that elevated circulating SM may increase susceptibility to OA. Using publicly available genome-wide association study (GWAS) summary statistics for total serum sphingomyelin levels (captured by large-scale proton nuclear magnetic resonance metabolomic profiling), we evaluated this hypothesis through bidirectional MR and colocalization analyses across four OA phenotypes. This investigation was complemented by an exogenous SM intervention in rats with DMM-induced OA, with joint pathology assessed by histology, micro-CT, and molecular assays. Taken together, the MR analyses evaluated whether genetically elevated circulating SM increases OA susceptibility, while the DMM experiment assessed whether exogenous SM influences disease progression after mechanical joint injury.

## 2. Materials and Methods

### 2.1. Study Design

This study integrated genetic analyses with an animal experiment. We used bidirectional two-sample MR and colocalization analyses of publicly available GWAS summary statistics to examine the relationship between serum SM and OA. We then administered exogenous SM to rats with DMM and assessed articular cartilage, subchondral bone microarchitecture, and OA-related molecular markers.

### 2.2. GWAS Data Sources

Summary-level GWAS data for serum SM were retrieved from the GWAS Catalog under accession number GCST90501272. The exposure phenotype was total circulating sphingomyelins (SMs), quantified using proton nuclear magnetic resonance (^1^H-NMR) spectroscopy and standardized before GWAS analysis. This dataset comprised 450,015 UK Biobank participants, including 434,646 individuals of British European ancestry, 8796 of Central/South Asian ancestry, and 6573 of African ancestry. After quality control, 10,413,938 imputed SNPs were available for analysis.

FinnGen Release 12 provided summary statistics for four OA phenotypes: overall OA (M13_ARTHROSIS; 101,454 cases), knee OA (M13_ARTHROSIS_KNEE; 61,356 cases), hip OA (M13_ARTHROSIS_COX; 30,802 cases), and other OA (M13_ARTHROSIS_OTH; 29,554 cases). These analyses shared a control group of 315,115 participants [24]. FinnGen participants were predominantly of European ancestry. No sample overlap between the exposure and outcome GWASs was identified.

### 2.3. Genetic Instrument Selection

Candidate instruments were SNPs associated with serum SM at genome-wide significance (*p* < 5 × 10^−8^). Quality control excluded duplicate and non-biallelic variants, as well as those lacking β coefficients, standard errors (SEs), effect allele frequencies (EAFs), or *p* values. Instrument strength was assessed using the F statistic, calculated as β^2^/SE^2^. Variants were retained if their EAF was between 0.01 and 0.99 and their F statistic exceeded 30.

Given differences in ancestry between the exposure and outcome GWASs and potential mismatch with external LD reference panels, we used a prespecified physical-distance clustering strategy. Each chromosome was divided into consecutive, non-overlapping 10-Mb windows, and the SNP most strongly associated with serum SM in each window was retained based on genomic position. This approach reduced reliance on external LD reference data and generated an approximately independent instrument set without directly estimating pairwise LD (Supplementary Data S1).

### 2.4. Two-Sample MR and Sensitivity Analyses

Before MR estimation, exposure and outcome data were harmonized to align effect estimates to the same effect allele. Effect allele frequency information was used to resolve palindromic variants where possible, and variants with unresolved allele orientation were excluded. Steiger directionality testing was performed to assess whether each instrument explained more variance in serum SM than in the corresponding OA outcome.

The IVW method served as the primary approach for estimating causal effects [25], and weighted median, MR-Egger regression, and weighted mode analyses were conducted as complementary methods. Effect estimates were reported as odds ratios (ORs) with 95% confidence intervals (CIs) per 1-standard-deviation increase in genetically predicted serum SM. Cochran’s Q test and the MR-Egger intercept test were used to assess between-SNP heterogeneity and directional horizontal pleiotropy, respectively [26,27]. Leave-one-out analysis was also performed by sequentially omitting each instrument and recalculating the IVW estimate to assess whether the overall association was driven by an individual variant. As a prespecified sensitivity analysis, the Wald ratio (β_outcome/β_exposure) was calculated for each instrument. Variants with an absolute Wald ratio greater than 2 were excluded and the MR analyses were repeated using the remaining instruments to assess the influence of potentially extreme single-SNP estimates (Supplementary Data S2).

For outcomes exhibiting significant between-SNP heterogeneity, two additional sensitivity analyses were prespecified. First, MR analyses were repeated after excluding potential heterogeneity-driving instruments, defined using either of two criteria: SNPs in the highest 10% of individual Q-statistic contributions or SNPs whose Q contributions had a false discovery rate (FDR) below 0.05. Second, instruments were stratified according to quartiles of their F statistics, and MR estimates were compared across strata to assess whether the findings were disproportionately driven by instruments of differing strength (Supplementary Data S3 and S4).

### 2.5. Reverse MR and Colocalization Analyses

Reverse MR followed the same general analytical framework as the forward analysis, except that genome-wide significant SNPs for overall, knee, hip, and other OA served as instruments and serum SM as the outcome. Instrument quality control, exposure–outcome harmonization, directionality assessment, and sensitivity analyses followed the principles described for the forward MR analyses. Because standardized serum SM was the outcome, reverse MR estimates were reported as β coefficients with 95% CIs (Supplementary Data S5 and S6).

To assess whether serum SM and OA might share a local causal variant, we applied coloc (v5.2.3) [28]. For each SM-associated lead SNP, variants located within a ±500-kb window were extracted for both GWAS datasets and harmonized across traits. Regions with fewer than 50 harmonized SNPs were excluded. The coloc algorithm calculates posterior probabilities for five hypotheses (H0–H4), where PP.H4 represents the posterior probability that both traits are associated and share a single causal variant within the region. Prior probabilities were set to p1 = 1 × 10^−4^, p2 = 1 × 10^−4^, and p12 = 1 × 10^−5^. PP.H4 > 0.80 was considered relatively strong evidence that serum SM and the corresponding OA phenotype shared a local causal variant.

Protein-coding genes within regions showing relatively strong colocalization evidence (PP.H4 > 0.80) were annotated using Ensembl and standardized according to HGNC gene nomenclature. Candidate genes were prioritized by keyword searches related to lipid metabolism and OA pathobiology. Gene prioritization was considered exploratory, and the identified candidates require subsequent functional validation (Supplementary Data S7 and S8).

### 2.6. Animals and Ethical Approval

Eight-week-old male Sprague-Dawley rats weighing 220 ± 20 g were obtained from the Laboratory Animal Center of Lanzhou University, maintained under specific pathogen-free conditions with free access to food and water and acclimated for 7 days before experimentation.

Animals were randomly assigned to the four experimental groups using a computer-generated random number sequence, with allocation concealment maintained through numbered, opaque envelopes prepared by an investigator not involved in surgery or outcome assessment. They were randomly assigned to four groups (*n* = 6 per group): sham surgery plus vehicle (Sham + Veh), DMM plus vehicle (DMM + Veh), DMM plus SM (DMM + SM), and sham surgery plus SM (Sham + SM). The group size was prespecified with reference to commonly used sample sizes in DMM-induced OA studies and the principle of minimizing animal use while retaining an appropriate experimental design [29,30,31]. Predefined exclusion criteria included postoperative infection, surgical complications, or death during the experimental period; no animals met these criteria, and all animals completed the study. Histological scoring, micro-CT analysis, and molecular assays were performed by investigators blinded to group allocation; statistical analysis was conducted on de-identified data. However, investigators performing surgery and administering injections could not be blinded due to the nature of these interventions. All procedures complied with relevant guidelines for the care and use of laboratory animals and were approved by the Animal Ethics Committee of the First Hospital of Lanzhou University (approval No. LDYYLL2026-673).

### 2.7. DMM Surgery and SM Administration

We established a DMM-induced OA model by transecting the medial meniscotibial ligament of the left knee; sham-operated rats underwent arthrotomy alone [32]. All animals then received the same standardized postoperative analgesic regimen. SM (CAS No. 383907-87-7; catalog No. S11408; purity ≥ 98%; Psaitong, Tianjin, China) was solubilized with 1% Tween 80 and 1% absolute ethanol, added dropwise into a predetermined volume of normal saline, and brought to the final volume with normal saline to obtain a clear solution. Beginning 72 h after surgery, SM-treated rats received SM via tail vein injection at 5 mg/kg every other day for 6 weeks. The dose, administration route, and dosing interval were prespecified on the basis of preliminary dose-ranging and tolerability assessments, with the aim of providing repeated systemic exposure while maintaining acceptable procedural tolerability. Vehicle-treated rats received an equivalent volume of vehicle via the same route and schedule. At the end of treatment, abdominal aortic blood was collected for serum isolation, along with the knee joints on the operated side for subsequent analyses.

### 2.8. Serum SM Measurement and Histologic Assessment

Serum SM quantification concentrations were measured using a commercial enzyme-linked immunosorbent assay kit (FineTest, Wuhan, China; catalog No. EU2663) in accordance with the manufacturer’s instructions. Serum samples were diluted 1:10 with the sample diluent supplied with the kit and assayed in triplicate. Absorbance was measured at 450 nm, and SM concentrations were determined using a standard curve covering 3.125–200 ng/mL. The manufacturer-reported assay sensitivity was 1.875 ng/mL. Intra-assay coefficients of variation were 5.43%, 4.97%, and 5.44%; inter-assay coefficients of variation were 5.01%, 5.39%, and 5.29%. According to the manufacturer’s documentation, the assay is specific for SM and has no reported significant cross-reactivity with related sphingolipids.

Knee joints were fixed in 4% paraformaldehyde for 48 h, decalcified in 10% ethylenediaminetetraacetic acid (EDTA; pH 7.4) for 4 weeks, embedded in paraffin, and sectioned at 4 μm. Three non-adjacent sections per joint, spaced 100 μm apart and spanning the central weight-bearing regions of the medial femoral condyle and tibial plateau, were stained with hematoxylin and eosin or Safranin O–Fast Green. Cartilage damage in the medial femoral condyle and tibial plateau was assessed using the Mankin and Osteoarthritis Research Society International (OARSI) scoring systems. Two trained observers, both blinded to group allocation, independently scored all sections without discussion or consensus procedures [33].

### 2.9. Micro-CT

Following fixation in 4% paraformaldehyde for 48 h, the knee joints underwent micro-computed tomography scanning using an NMC-200 system at a tube voltage of 80 kV and a tube current of 0.06 mA. Images were acquired with a voxel size of 0.051 mm × 0.051 mm × 0.051 mm, corresponding to isotropic voxels. Image reconstruction was performed using the filtered back-projection (FDK) algorithm, and the reconstructed images were analyzed using Avatar three-dimensional analysis software (v3.0). The CT threshold settings were 850/700, as recorded in the micro-CT experimental report, and were applied consistently during image analysis.

The subchondral bone ROI was delineated in Avatar software as the trabecular bone extending from the metaphyseal–epiphyseal junction distally in the femur and proximally in the tibia, beneath the weight-bearing medial articular surface. Within the predefined ROI, we quantified bone mineral density (BMD), bone volume fraction (BV/TV), connectivity density (Conn.D), structure model index (SMI), trabecular number (Tb.N), trabecular thickness (Tb.Th), trabecular separation (Tb.Sp), and trabecular pattern factor (Tb.Pf) [34].

### 2.10. RT-qPCR

We isolated total RNA from articular cartilage, determined its concentration and purity, and synthesized complementary DNA by reverse transcription. Subsequent RT-qPCR analysis quantified the relative transcript abundance of *Mmp13*, *IL-1β*, *Smpdl3a*, and *Cd36*, with *β-actin* serving as the reference gene. Relative expression was calculated using the 2^−ΔΔCt^ method [35] (Supplementary Table S1).

### 2.11. Western Blotting

Following extraction from articular cartilage, total protein concentration was determined before sodium dodecyl sulfate-polyacrylamide gel electrophoresis and transfer to membranes. After blocking, we incubated the membranes with primary antibodies against MMP13, cleaved caspase-3, phosphorylated p38, total p38, and β-actin, followed by incubation with horseradish peroxidase-conjugated secondary antibodies. Chemiluminescence visualized the protein bands, and ImageJ (v1.53) enabled densitometric quantification. β-actin served as the loading control (Supplementary Table S2).

### 2.12. Statistical Analysis

MR and colocalization analyses used R version 4.6.0. Animal data were analyzed using SPSS v23.0 and R v4.6.0. Continuous variables are expressed as the mean ± standard deviation. For the 2 × 2 factorial design (Surgery: Sham vs. DMM; Treatment: Vehicle vs. SM), two-way analysis of variance (ANOVA) assessed main effects and the Surgery × Treatment interaction. Intraclass correlation coefficients (ICCs) for histological scoring were calculated using a two-way random-effects model with absolute agreement. *p* < 0.05 was considered statistically significant. For animal experiments, statistical significance in figures is denoted as: * *p* < 0.05, ** *p* < 0.01, *** *p* < 0.001 for Sham + Veh versus DMM + Veh(or Sham + Veh versus Sham + SM in ELISA); # *p* < 0.05, ## *p* < 0.01, ### *p* < 0.001 for DMM + Veh versus DMM + SM.

## 3. Results

### 3.1. Forward MR Analysis

A total of 178 SNPs were selected as instrumental variables for serum SM using the prespecified 10-Mb physical-distance clustering strategy; all had F statistics > 30, with a mean F statistic of 1407.2.

**Figure 1:**
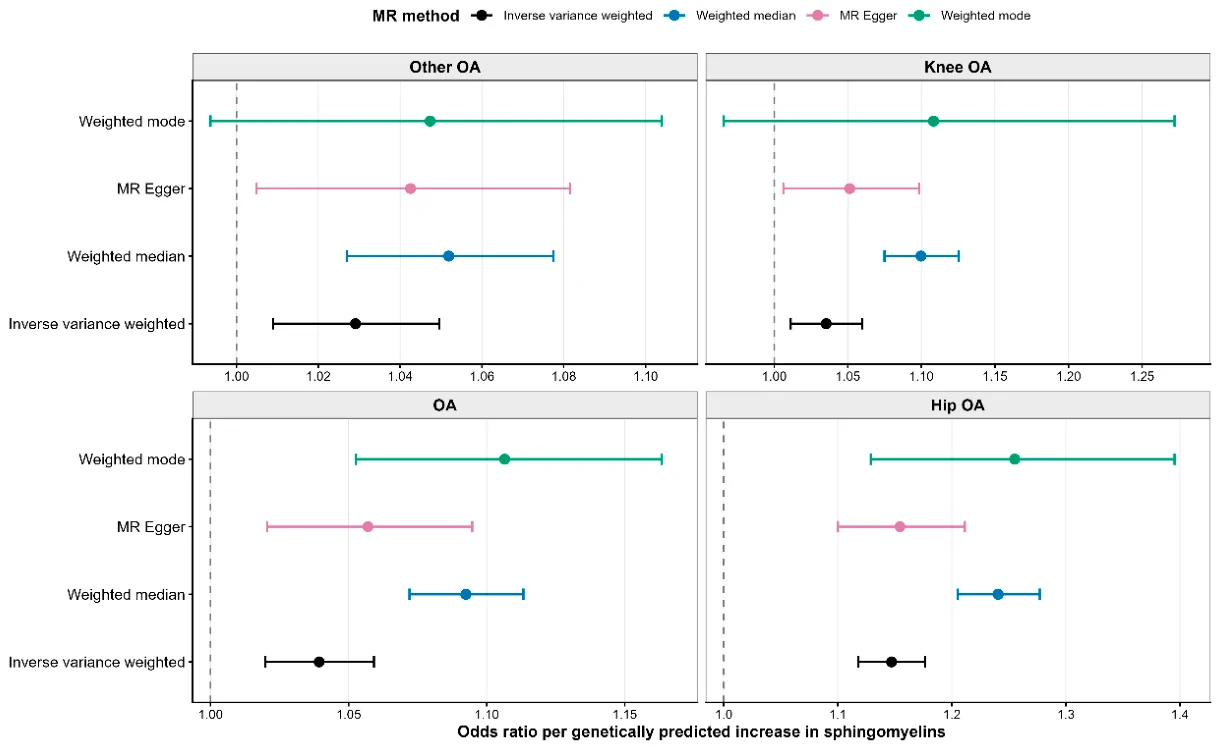
Associations of genetically predicted serum SM with OA. Forest plots show ORs and 95% CIs per 1-SD increase in genetically predicted serum SM.

In the primary IVW analyses, each 1-SD increase in genetically predicted serum SM was associated with higher odds of overall OA (OR, 1.039; 95% CI, 1.020–1.059; *p* < 0.001), hip OA (OR, 1.147; 95% CI, 1.118–1.177; *p* < 0.001), knee OA (OR, 1.035; 95% CI, 1.011–1.060; *p* = 0.004), and other OA (OR, 1.029; 95% CI, 1.009–1.050; *p* = 0.004). The weighted-median and MR-Egger estimates were directionally consistent with the IVW estimates across all four OA phenotypes, with ORs ranging from 1.052 to 1.241 for the weighted-median method and from 1.043 to 1.155 for the MR-Egger method. The largest IVW OR was observed for hip OA (Supplementary Data S1 and S2; Figure 1).

### 3.2. Sensitivity and Heterogeneity Analyses

Cochran’s Q tests indicated evidence of heterogeneity for overall OA (Q = 458.949; *p* < 0.01; I^2^ = 62.3%), knee OA (Q = 487.729; *p* < 0.01; I^2^ = 64.3%), and hip OA (Q = 313.793; *p* < 0.01; I^2^ = 46.1%), but not for other OA (Q = 194.499; *p* = 0.115; I^2^ = 11.6%). MR-Egger intercept tests provided no evidence of directional horizontal pleiotropy for overall OA, knee OA, hip OA, or other OA, respectively (*p* = 0.267, 0.758, 0.416, and 0.413).

Leave-one-out analyses showed that removal of the most influential SNP did not materially change the direction of the IVW estimates for overall OA (rs73015024 removed; OR, 1.042; 95% CI, 1.023–1.061), knee OA (rs1065853 removed; OR, 1.037; 95% CI, 1.013–1.062), hip OA (rs1065853 removed; OR, 1.152; 95% CI, 1.123–1.181), or other OA (rs73015024 removed; OR, 1.032; 95% CI, 1.013–1.052). Steiger filtering excluded none of the harmonized variants. Exclusion of SNPs with an absolute Wald ratio greater than 2 resulted in only minor changes in the estimates across the four OA phenotypes (Supplementary Data S2).

### 3.3. Exploratory Heterogeneity Analyses

In the prespecified exploratory analyses, exclusion of the 10% of SNPs with the largest contributions to Cochran’s Q yielded IVW estimates of OR, 1.044 (95% CI, 1.029–1.059) for overall OA; OR, 1.040 (95% CI, 1.021–1.060) for knee OA; OR, 1.170 (95% CI, 1.145–1.195) for hip OA; and OR, 1.035 (95% CI, 1.016–1.055) for other OA.

**Figure 2:**
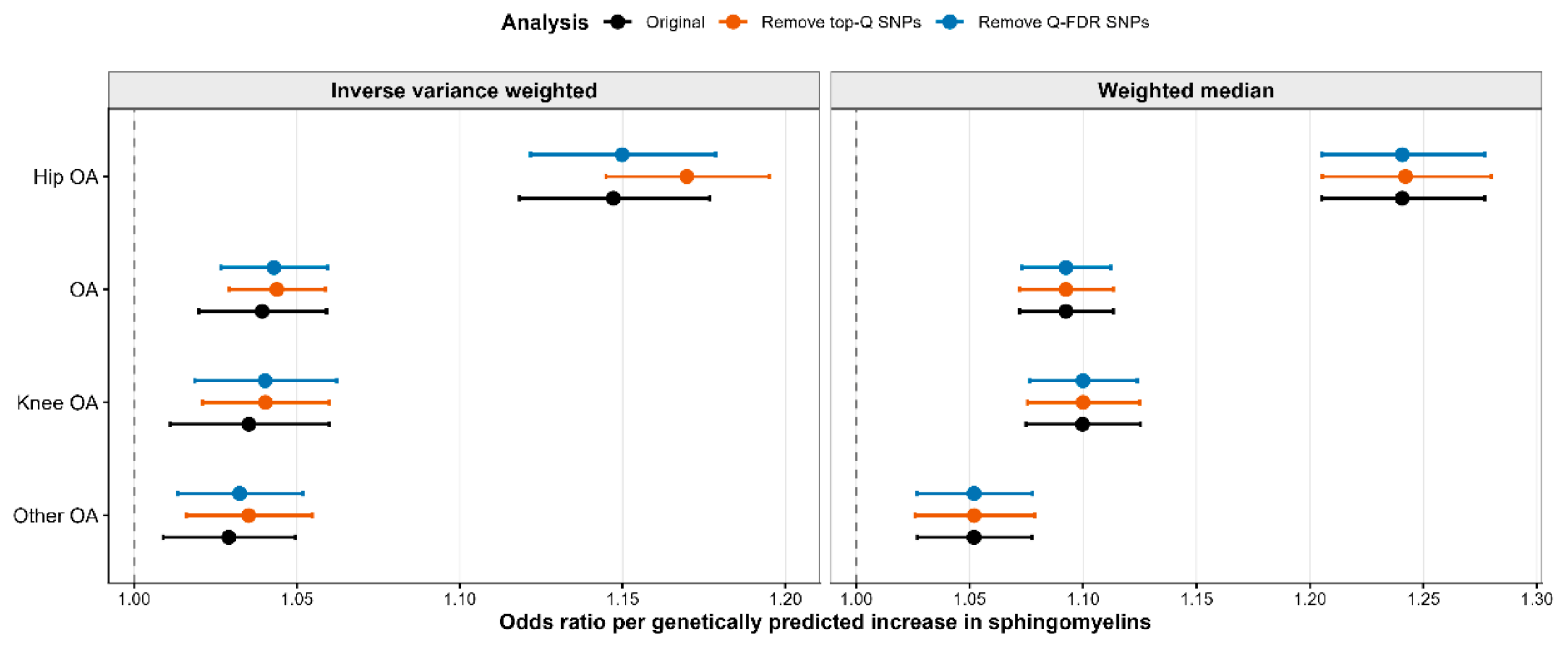
Sensitivity analyses addressing heterogeneity. MR estimates obtained using the original instrument set are compared with those derived after removing either the largest contributor to Cochran’s Q statistic or SNPs with FDR-significant Q contributions. Error bars indicate 95% CIs.

After exclusion of SNPs whose contributions to Cochran’s Q remained significant after false discovery rate correction, the corresponding IVW estimates were OR, 1.043 (95% CI, 1.027–1.059) for overall OA; OR, 1.040 (95% CI, 1.019–1.062) for knee OA; OR, 1.150 (95% CI, 1.122–1.179) for hip OA; and OR, 1.032 (95% CI, 1.013–1.050) for other OA. The direction of the associations was unchanged after both exclusion procedures. Heterogeneity was reduced, although to different extents, with I^2^ values after the top-10% and FDR-based exclusions of 32.0% and 45.1% for overall OA, 42.6% and 54.8% for knee OA, 16.7% and 42.7% for hip OA, and 0% under both procedures for other OA, respectively (Supplementary Data S3; Figure 2).

**Figure 3:**
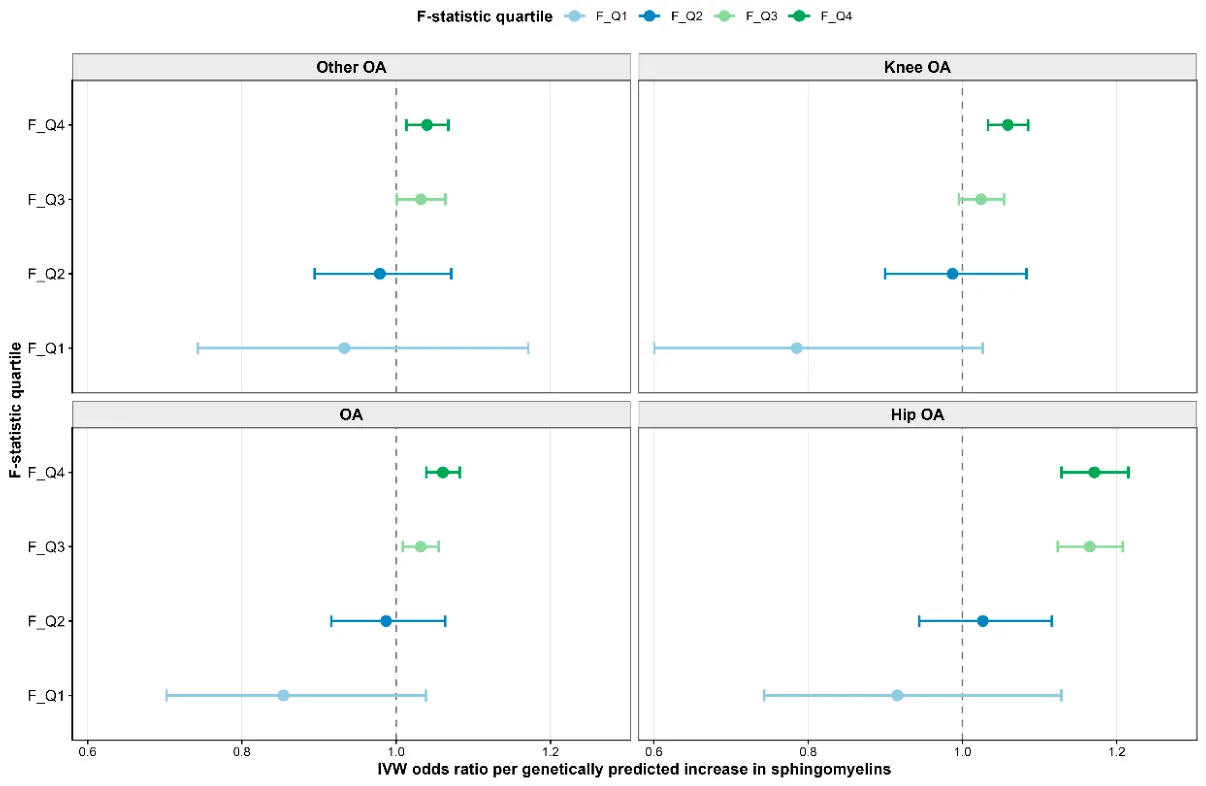
MR analyses stratified by instrument strength. Instruments were divided into quartiles according to their F statistics. Points and error bars represent IVW ORs and 95% CIs.

Stratification by F-statistic quartiles showed some variation in the estimates across instrument-strength strata. Estimates in the higher-F quartiles, F_Q3 and F_Q4, were generally more precise, whereas those in the lower-F quartiles, F_Q1 and F_Q2, had wider confidence intervals (Supplementary Data S4; Figure 3).

### 3.4. Reverse MR Analysis

Reverse MR provided no consistent evidence that genetic liability to OA was associated with serum SM levels. Using 52, 53, 31, and 17 instrumental variables for overall OA, knee OA, hip OA, and other OA, respectively, the primary IVW estimates were β, −0.043 (95% CI, −0.099 to 0.013; *p* = 0.13) for overall OA; β, −0.040 (95% CI, −0.084 to 0.005; *p* = 0.08) for knee OA; β, 0.011 (95% CI, −0.014 to 0.036; *p* = 0.39) for hip OA; and β, −0.031 (95% CI, −0.072 to 0.009; *p* = 0.13) for other OA. Although the weighted-median estimates indicated potential associations for knee OA and hip OA, the corresponding IVW and MR-Egger estimates did not provide consistent supporting evidence (Supplementary Data S5 and S6).

### 3.5. Colocalization Analysis

Among the 992 locus–phenotype comparisons that met the prespecified inclusion criteria, 21 had a posterior probability for H4 greater than 0.80, indicating relatively strong evidence that the serum SM and OA association signals within these regions might share a local causal variant. These included seven comparisons for knee OA, six for overall OA, six for hip OA, and two for other OA. By comparison, 144 locus–phenotype comparisons showed greater support for H3, under which both traits were associated within the region but with distinct causal variants.

**Figure 4:**
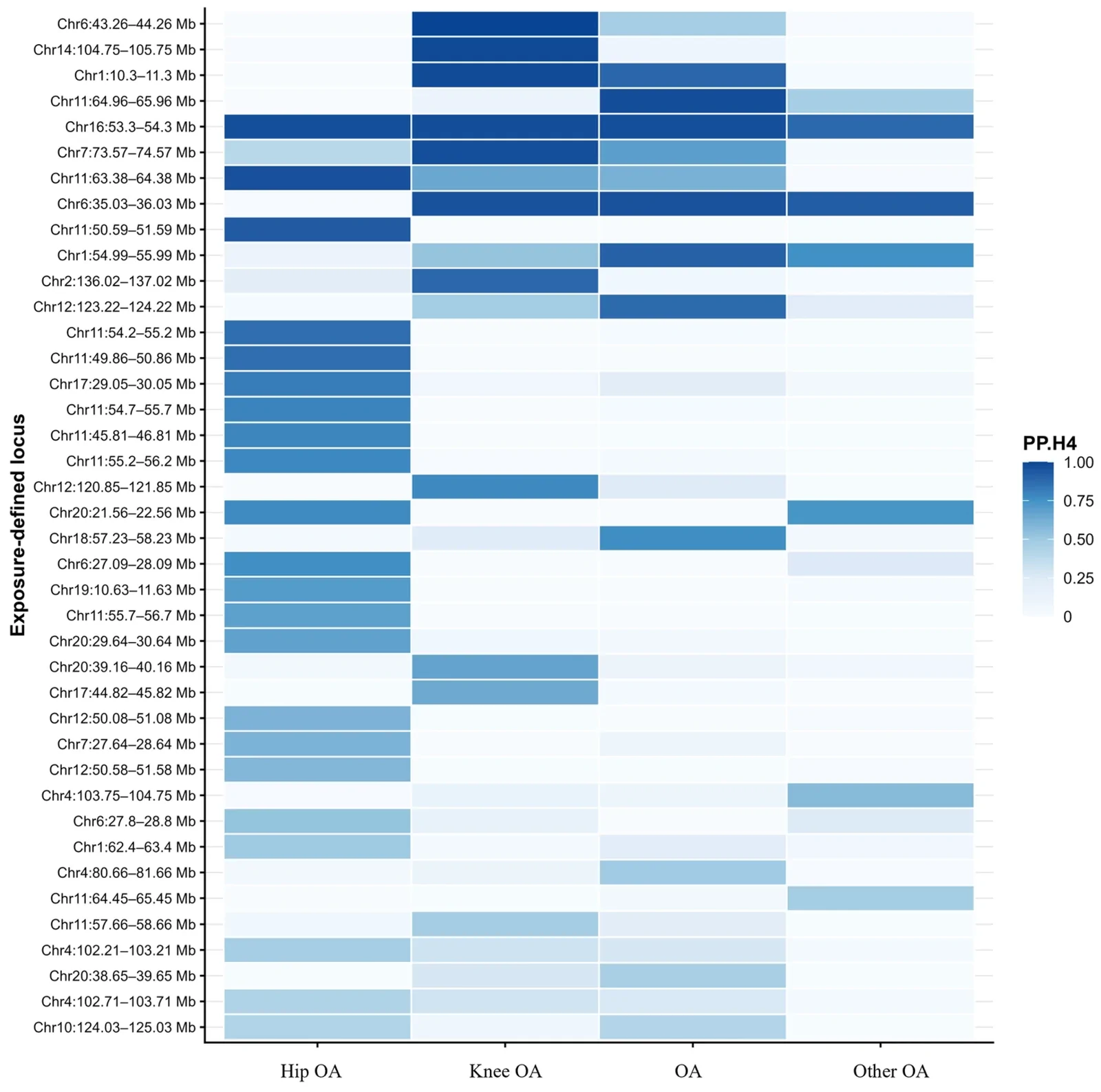
Colocalization of serum SM and OA. The heatmap shows the posterior probability of a shared causal variant (PP.H4) across exposure-defined loci and OA phenotypes. Darker shading denotes higher PP.H4 values and stronger evidence of colocalization; PP.H4 > 0.80 was considered strong evidence.

The distribution of colocalized regions varied across the four OA phenotypes. The regions with the highest PP.H4 values were chr6:43,256,169–44,256,169 for knee OA (PP.H4 = 0.999), chr11:64,956,685–65,956,685 for overall OA (PP.H4 = 0.974), chr16:53,297,908–54,297,908 for hip OA (PP.H4 = 0.969), and chr6:35,026,706–36,026,706 for other OA (PP.H4 = 0.917). Evidence supporting H4 was observed for multiple OA phenotypes at two regions: chr6:35,026,706–36,026,706 for knee OA, overall OA, and other OA, and chr16:53,297,908–54,297,908 for knee OA, overall OA, and hip OA (Supplementary Data S7; Figure 4).

Exploratory annotation of the colocalized regions identified BLTP3A, PNPLA1, TMEM61, PLCB3, MAJIN, TM7SF2, EFEMP2, and ILRUN as candidate genes. These genes were annotated to biological processes related to lipid metabolism, sphingolipid signaling, membrane organization, extracellular matrix regulation, or inflammation (Supplementary Data S8).

### 3.6. Serum SM Concentrations and Histological Assessment

Serum SM concentrations were measured by ELISA to confirm systemic exposure. In DMM rats, SM treatment increased serum SM concentrations from 104.1 ± 26.8 ng/mL in the DMM + Veh group to 200.5 ± 31.4 ng/mL in the DMM + SM group (mean difference, 96.4 ng/mL; simultaneous 95% CI, 57.9–134.8; Šídák-adjusted *p* = 7.2 × 10^−6^). Serum SM concentrations were also numerically higher in the Sham + SM group than in the Sham + Veh group (177.0 ± 21.8 vs. 92.1 ± 24.2 ng/mL).

**Figure 5:**
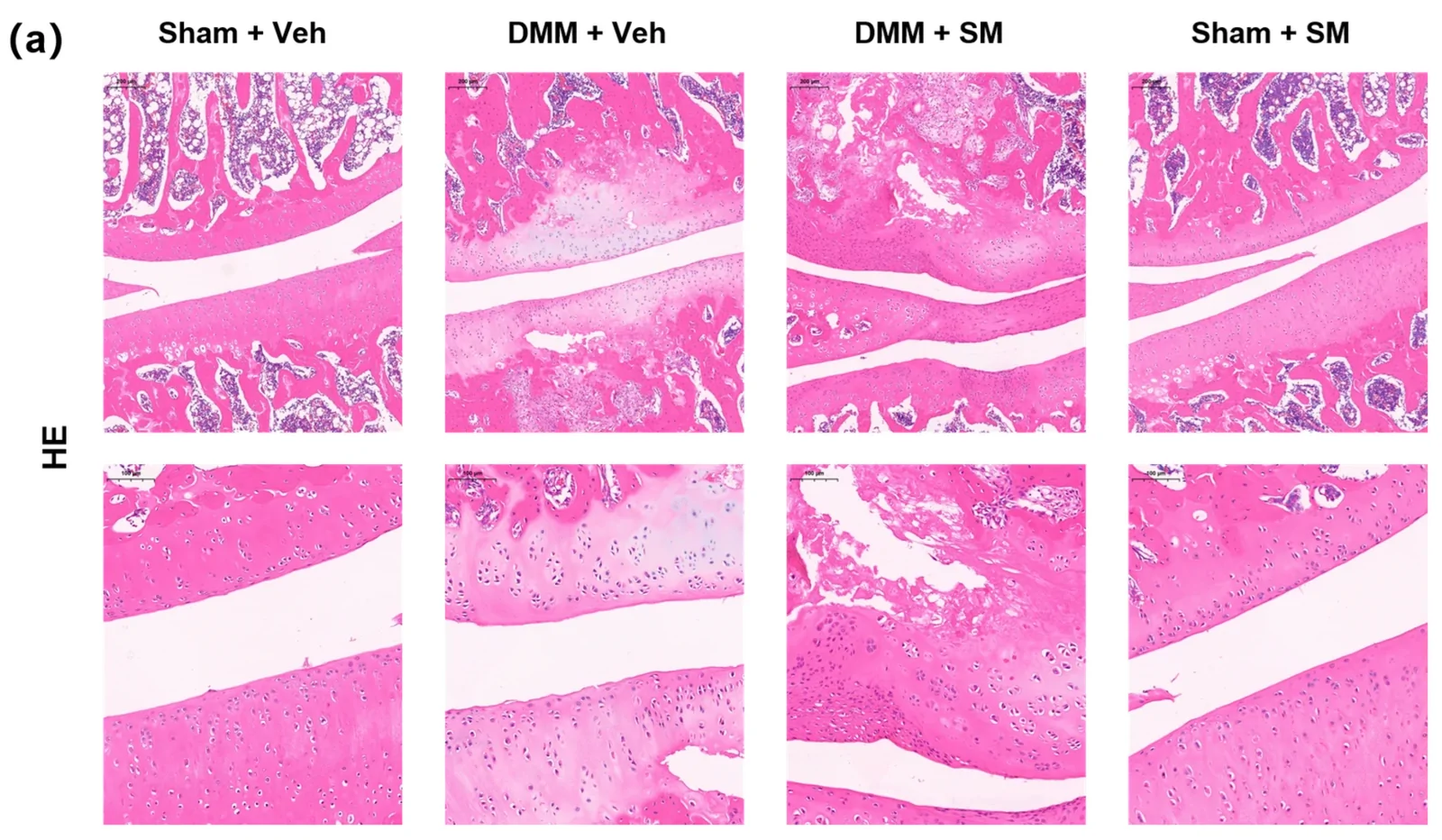

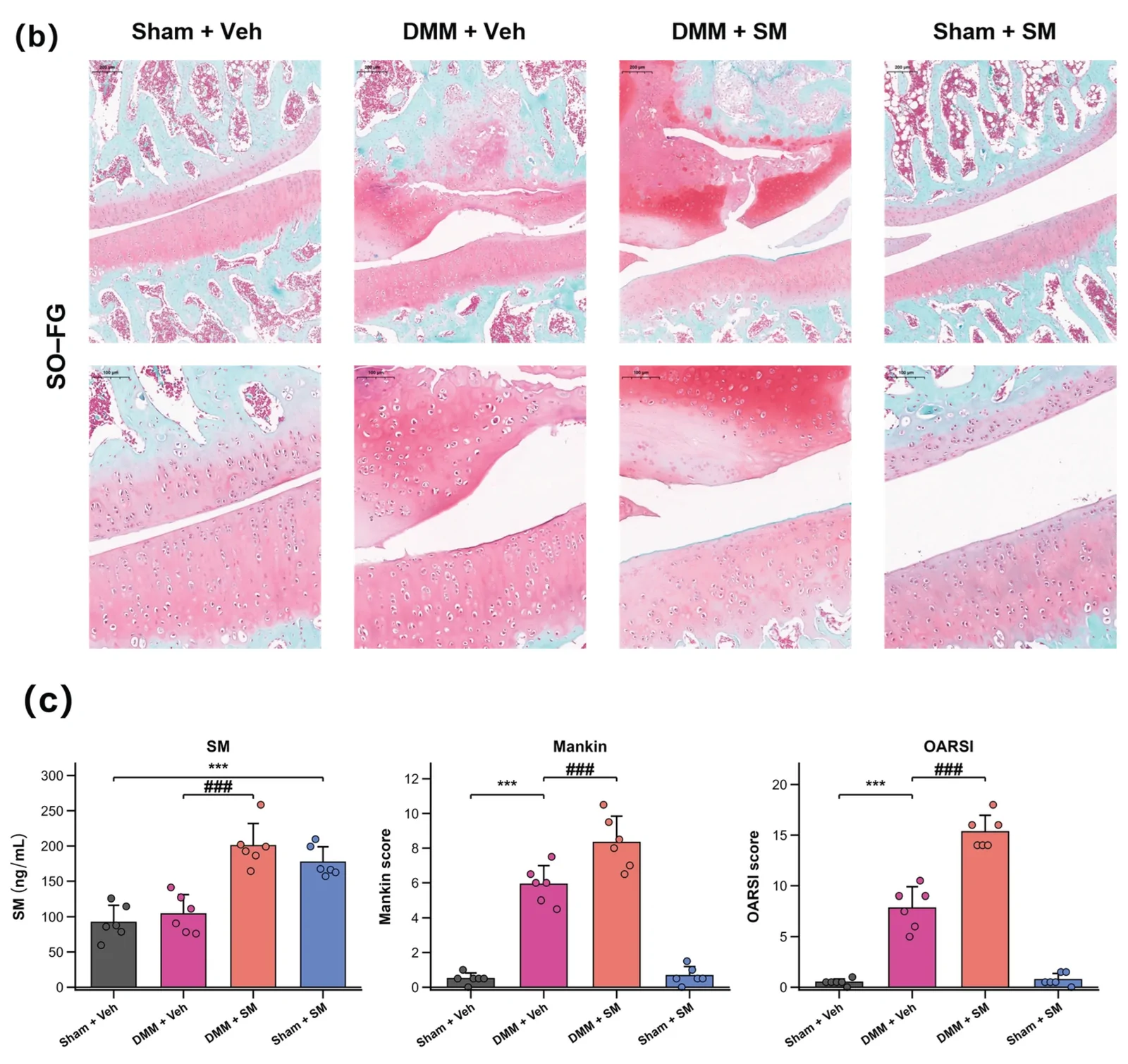
Exogenous sphingomyelin exacerbates DMM-induced cartilage degeneration. (**a**) Representative hematoxylin and eosin staining. (**b**) Representative Safranin O-Fast Green staining. (**c**) Serum sphingomyelin concentrations and Mankin and OARSI scores. Upper and lower images were acquired at ×160 and ×400 magnification, with scale bars of 200 and 100 μm, respectively. Data are mean ± SD (*n* = 6 per group). DMM, destabilization of the medial meniscus; OARSI, Osteoarthritis Research Society International; SM, sphingomyelin; Veh, vehicle. *** *p* < 0.001 for Sham + Veh versus DMM + Veh(or Sham + Veh versus Sham + SM in ELISA); ### *p* < 0.001 for DMM + Veh versus DMM + SM.

Articular cartilage damage was assessed using OARSI and Mankin scores at the animal level. Compared with the Sham + Veh group, DMM + Veh animals showed markedly higher OARSI and Mankin scores (7.83 ± 2.07 vs. 0.50 ± 0.32 and 5.92 ± 1.07 vs. 0.50 ± 0.32, respectively; both Šídák-adjusted *p* < 0.001). Within the DMM groups, SM treatment further increased both OARSI and Mankin scores. OARSI scores were 15.33 ± 1.63 in the DMM + SM group versus 7.83 ± 2.07 in the DMM + Veh group (Šídák-adjusted *p* < 0.001), while Mankin scores were 8.33 ± 1.51 versus 5.92 ± 1.07, respectively (Šídák-adjusted *p* < 0.001). Two-way ANOVA showed significant main effects of surgery and SM treatment, as well as significant surgery × SM treatment interactions, for both OARSI and Mankin scores (all *p* ≤ 0.0102) (Supplementary Tables S3 and S4, Figure 5).

Interobserver reliability was high for both OARSI and Mankin scores, based on a two-way random-effects, absolute-agreement ICC model (single-measure ICC(2,1) = 0.952, 95% CI 0.893–0.979, and 0.957, 95% CI 0.903–0.981, respectively; average-measure ICC(2,k) = 0.975 and 0.978, respectively).

### 3.7. Micro-CT Analysis of Subchondral Bone

Two-way ANOVA showed significant main effects of surgery and SM treatment on the eight subchondral bone parameters evaluated (BV/TV, Tb.Th, Tb.Sp, Tb.N, Tb.Pf, SMI, Conn.D, and BMD; all *p* < 0.001 for surgery and *p* < 0.05 for SM treatment). Significant surgery × SM interactions were observed for BV/TV (*p* = 0.030) and degree of anisotropy (DA; *p* = 0.008), whereas no significant interactions were detected for the remaining seven parameters (all *p* > 0.05).

**Figure 6:**
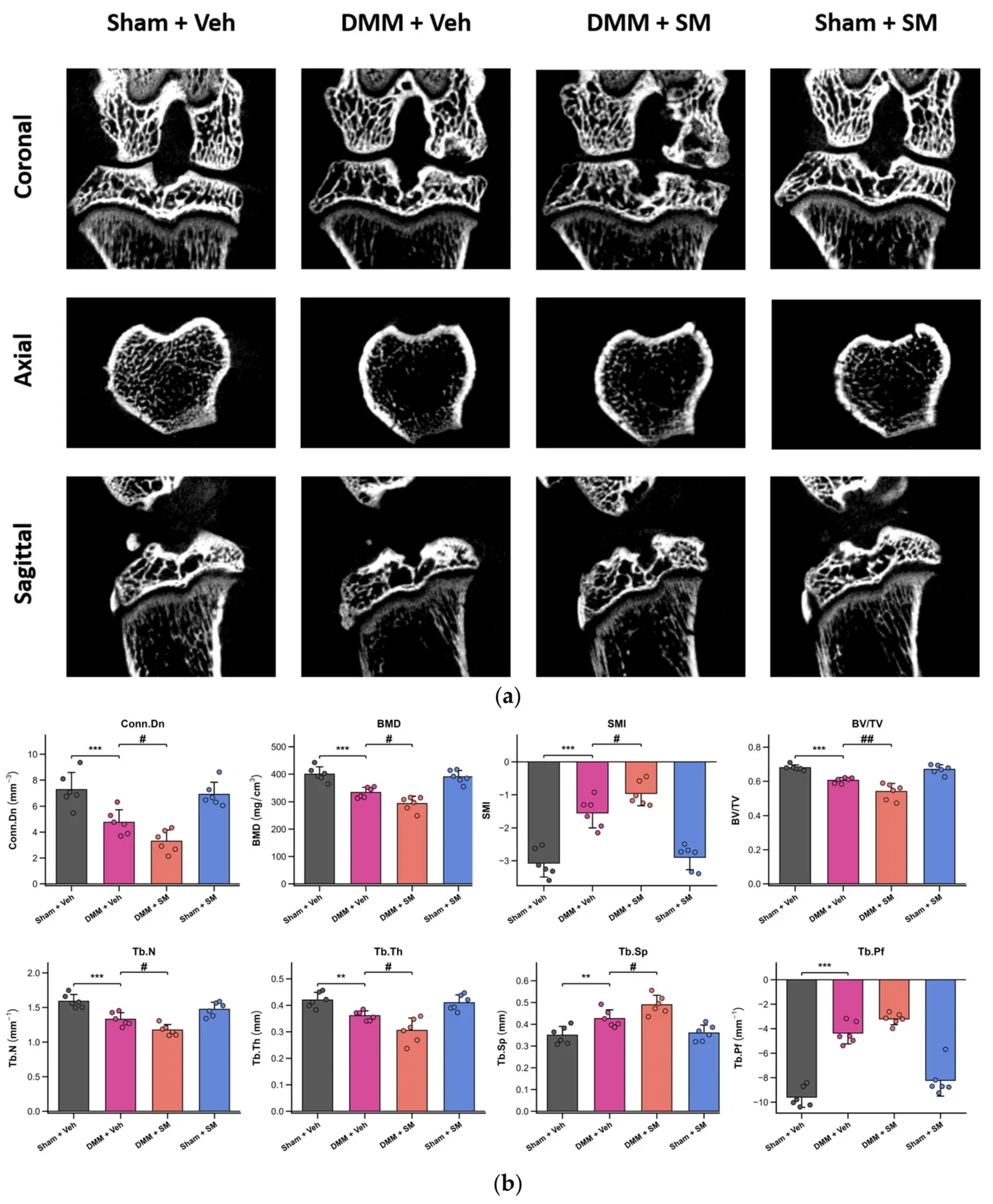
Subchondral bone microarchitecture in the experimental groups. (**a**) Representative coronal, axial, and sagittal micro-CT images. (**b**) Quantitative analysis of connectivity density (Conn.Dn), bone mineral density (BMD), structure model index (SMI), bone volume fraction (BV/TV), trabecular number (Tb.N), thickness (Tb.Th), separation (Tb.Sp), and pattern factor (Tb.Pf). Data are mean ± SD (*n* = 6 per group). ** *p* < 0.01, *** *p* < 0.001 for Sham + Veh versus DMM + Veh; # *p* < 0.05, ## *p* < 0.01 for DMM + Veh versus DMM + SM.

Šídák-adjusted comparisons showed that DMM + Veh animals had lower trabecular bone volume fraction, thickness, number, connectivity density, and mineral density, but higher trabecular separation, Tb.Pf, and SMI than Sham + Veh animals (all adjusted *p* < 0.01). In DMM-operated animals, SM treatment shifted these measures further in the same respective directions, with significant differences for all parameters except Tb.Pf (adjusted *p* < 0.05 for the other seven parameters; adjusted *p* = 0.079 for Tb.Pf) (Supplementary Tables S3 and S4; Figure 6).

### 3.8. qPCR and Western Blot Analysis

**Figure 7:**
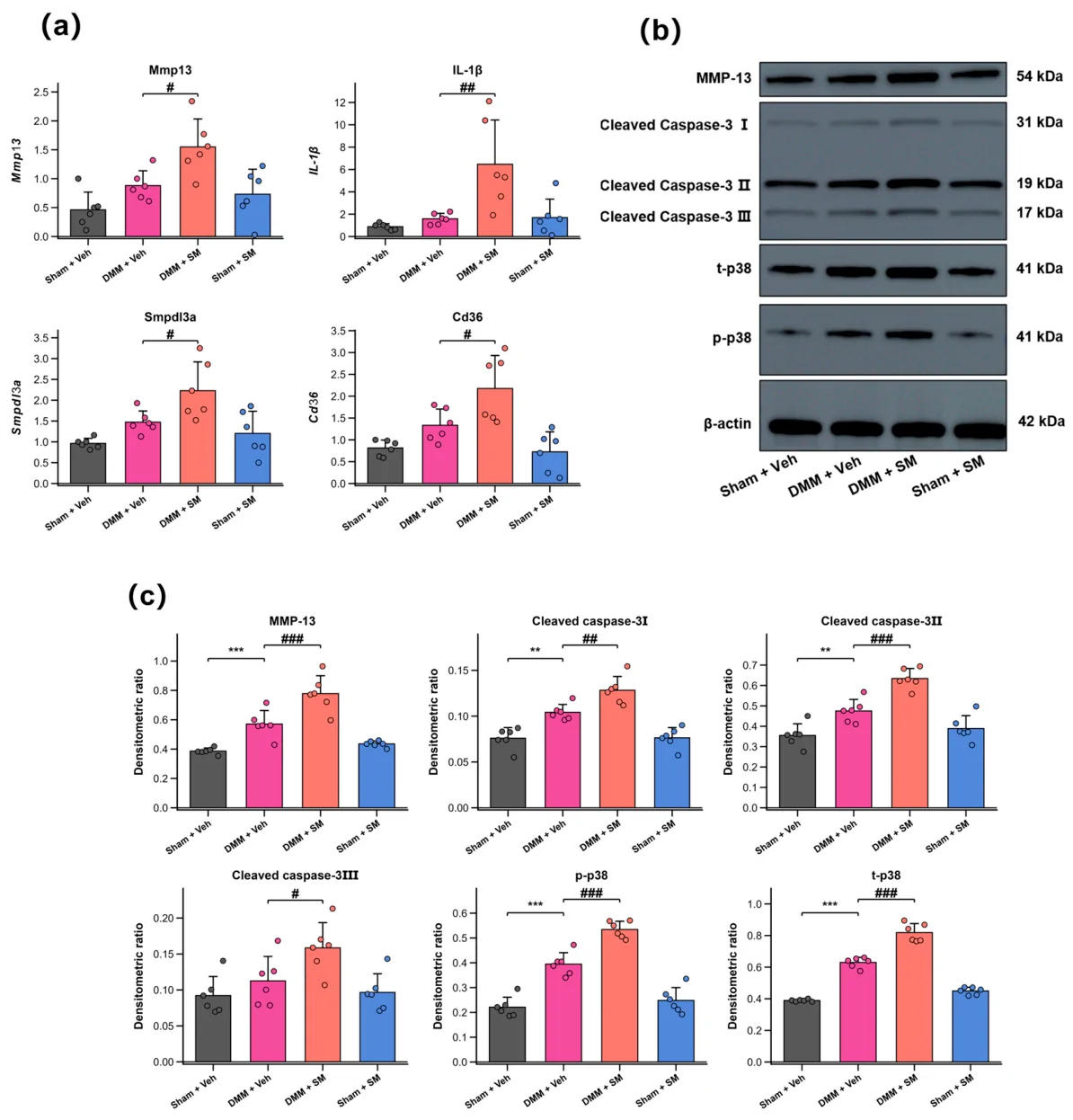
Exogenous SM-induced molecular changes in articular cartilage. (**a**) Relative cartilage mRNA expression of Mmp13, IL-1β, Smpdl3a, and Cd36, quantified by RT-qPCR and normalized to β-actin. (**b**) Western blot analysis of MMP13, total p38, phosphorylated p38, cleaved caspase-3, and β-actin. (**c**) Densitometric quantification of the indicated Western blot bands, normalized to β-actin. Data are mean ± SD (*n* = 6 per group). ** *p* < 0.01, *** *p* < 0.001 for Sham + Veh versus DMM + Veh; # *p* < 0.05, ## *p* < 0.01, ### *p* < 0.001 for DMM + Veh versus DMM + SM.

Two-way ANOVA showed significant main effects of surgery on all four qPCR outcomes: *Mmp13* (F_1,20_ = 15.879, *p* = 7.29 × 10^−4^), *Il1b* (F_1,20_ = 9.597, *p* = 0.00567), *Smpdl3a* (F_1,20_ = 16.751, *p* = 5.66 × 10^−4^), and *Cd36* (F_1,20_ = 24.429, *p* = 7.84 × 10^−5^). SM treatment also significantly affected *Mmp13*, *Il1b*, and *Smpdl3a* expression, whereas its main effect on *Cd36* was not significant. Significant surgery-by-treatment interactions were observed for *Il1b* and *Cd36*, but not for *Mmp13* or *Smpdl3a*. Within the DMM-operated animals, SM treatment increased the expression of all four genes compared with vehicle treatment, including *Mmp13, Il1b, Smpdl3a,* and *Cd36* (adjusted *p* = 0.0129, 0.00175, 0.0197, and 0.0136, respectively) (Supplementary Tables S3 and S4; Figure 7).

At the protein level, surgery and SM treatment exerted significant main effects on MMP-13, cleaved caspase-3 at 31 and 19 kDa, total p38, and phosphorylated p38. Surgery also affected the 17-kDa cleaved caspase-3 band and the phosphorylated-to-total p38 ratio, whereas the corresponding main effects of SM treatment were not significant. Significant surgery-by-treatment interactions were detected for MMP-13, cleaved caspase-3 at 31 and 19 kDa, total p38, and phosphorylated p38, but not for the 17-kDa cleaved caspase-3 band or the phosphorylated-to-total p38 ratio.

In DMM-operated animals, SM treatment increased MMP-13 and cleaved caspase-3 protein levels relative to vehicle treatment, with significant differences observed for the 31-, 19-, and 17-kDa cleaved caspase-3 bands (all *p* < 0.05). Total p38 and phosphorylated p38 levels were also increased after SM treatment (adjusted *p* = 1.67 × 10^−8^ and 3.62 × 10^−5^, respectively), whereas the phosphorylated-to-total p38 ratio remained unchanged (adjusted *p* = 0.836) (Supplementary Tables S3 and S4; Figure 7).

## 4. Discussion

Cartilage homeostasis depends on coordinated energy metabolism and membrane lipid regulation, both of which are disrupted by aging, obesity, and inflammation [16,36]. Sphingomyelin (SM), a major membrane sphingolipid, participates in membrane organization, lipid signaling, and cellular stress responses, potentially linking systemic metabolic alterations to joint pathology [37,38,39]. Using Mendelian randomization, genetic colocalization, and a surgically induced rat OA model, this study evaluated the relationship between serum SM and OA across population genetic, locus-specific, and in vivo phenotypic levels and found supportive evidence for an association between elevated serum SM and OA susceptibility. Mendelian randomization leverages genetic variants as instrumental variables to infer causal direction while minimizing confounding. Forward MR using 178 serum SM instruments (mean F-statistic 1407.2) indicated that genetically predicted higher SM is associated with increased risk across overall OA, hip OA, knee OA, and other OA, with effect estimates ranging from OR 1.029 to 1.147. Reverse MR provided no consistent evidence that OA liability altered SM levels. Multiple sensitivity analyses, including weighted median, MR-Egger, leave-one-out, and exclusion of outlier SNPs, yielded directionally consistent results, supporting the robustness of the primary findings. These results support the hypothesis that circulating SM may contribute to OA susceptibility. The modest effect sizes and heterogeneity across instrumental variants likely reflect the influence of multiple genetic pathways, residual pleiotropy from correlated lipid metabolites, and ancestral diversity between the exposure and outcome cohorts. The physical-distance clustering strategy used to define independent instruments may also introduce variability relative to ancestry-matched LD-based approaches. Nonetheless, the directionality and consistency of associations across joint sites and sensitivity frameworks position MR as supportive evidence for a potential causal relationship between serum SM and OA risk. To further evaluate whether this relationship reflects shared genetic architecture at specific genomic loci, we conducted colocalization analysis [40,41,42]. Colocalization analysis tested whether SM and OA share causal variants within the same genomic regions [28,43,44,45]. Of 992 region–phenotype comparisons, 21 showed PP.H4 > 0.80, indicating that SM and the corresponding OA phenotype are likely driven by a shared variant in those loci; an additional 144 favored distinct variants within the same region. The 21 shared-signal loci span overall, knee, hip, and other OA subtypes, implicating genes involved in lipid transport (BLTP3A, PNPLA1), sphingolipid homeostasis (TMEM61, SMPDL3A), membrane organization (MAJIN, TM7SF2), extracellular matrix regulation (EFEMP2), and inflammatory signaling (ILRUN, PLCB3). This locus-level convergence supports further functional interrogation of candidate genes and the pathways they regulate. The rat DMM experiments evaluated SM exposure in an in vivo setting. Histological and micro-CT analyses showed that SM treatment exacerbated cartilage erosion, proteoglycan loss, and subchondral bone deterioration in DMM animals, with significant main effects of surgery and SM treatment and interaction effects observed for selected outcomes including OARSI score, Mankin score, BV/TV, and DA. To explore the molecular basis of these structural changes, we examined cartilage gene and protein expression. Molecular profiling revealed corresponding changes: surgery and SM treatment elevated *Mmp13*, *Il1b*, *Smpdl3a*, and *Cd36* transcripts, with interactions observed for *Il1b* and *Cd36*; DMM + SM animals showed further increases in MMP-13, cleaved caspase-3, total p38, and phosphorylated p38 protein levels. These molecular responses align functionally with the observed structural pathology. Elevated *Cd36* and *Smpdl3a* are consistent with altered lipid handling [46,47]; increased *Il1b* and *Mmp13* reflect inflammatory and matrix-degradative activity [48,49]; cleaved caspase-3 elevation indicates apoptotic stress; and the coordinate rise in total and phosphorylated p38 suggests increased p38-pathway protein abundance [50,51]. Notably, the most pronounced structural and molecular changes occurred when mechanical destabilization and SM treatment were combined, suggesting a potential interaction between joint injury and metabolic stress. This interaction pattern supports the hypothesis that elevated SM may amplify joint pathology under mechanical instability—a “mechanical injury–metabolic amplification” framework. Some between-group differences did not reach statistical significance after correction, and not all parameters exhibited interaction effects, likely reflecting limited sample size, variability in surgical destabilization, and incomplete recapitulation of human OA pathophysiology. Nonetheless, the directional alignment between genetic predictions and in vivo phenotypes strengthens the evidence linking SM to OA susceptibility. This study integrated population genetics, locus-level colocalization, and experimental phenotyping to evaluate the SM–OA relationship. MR analysis supported a directional association and reduced concern about reverse causation. Colocalization identified select genomic regions where SM and OA share genetic signals. Animal experiments provided in vivo evidence that SM treatment worsens cartilage and bone pathology in the context of joint injury, accompanied by molecular changes in lipid-handling, inflammatory, matrix-degradative, and stress-signaling pathways. The convergence of these complementary lines of evidence—spanning genetic inference, locus-level overlap, structural phenotypes, and molecular responses—positions SM as a promising candidate metabolic factor in OA susceptibility and a mechanistic entry point for further investigation. Several limitations merit consideration. Regarding genetic analysis, MR instrumental variables represent genetically predicted total serum SM, lacking resolution for specific molecular species, lipoprotein carriers, or tissue distribution. Residual pleiotropy from correlated lipid pathways—ceramides, cholesterol, and triglycerides—cannot be fully excluded. Ancestry composition differs between the exposure and outcome cohorts, and the physical-distance clustering strategy used here does not equate to ancestry-matched LD clumping. Colocalization findings depend on model assumptions and variant coverage; while PP.H4 > 0.80 supports shared local signals, it does not pinpoint functional genes or define regulatory direction. Regarding animal experiments, exogenous SM was administered intravenously in a surgically destabilized joint, contrasting with lifelong genetic variation in circulating SM and the gradual pathophysiology of human OA. Only male rats were studied, and serum ELISA confirmed systemic exposure without resolving tissue-level SM concentrations or molecular species composition. Regarding molecular interpretation, markers show coordinated stress responses, yet their temporal ordering and causal interdependencies remain unresolved. These limitations primarily affect interpretation of specific SM species, tissue sites of action, and downstream mechanisms; they do not alter the directional consistency across genetic, locus-level, and phenotypic evidence. The convergent findings warrant further mechanistic investigation to clarify the role of SM in OA pathogenesis. Future work should clarify SM molecular specificity, tissue localization, and causal pathways across three complementary levels. Population genetic and lipidomic studies can distinguish individual SM species, stratify by carrier and tissue distribution, and employ multivariable approaches to isolate SM effects from correlated lipid pathways. Tissue and cellular mechanistic studies—integrating joint transcriptomics, chromatin mapping, lipidomics, and cell-type-specific perturbation—will determine whether circulating SM changes correspond to local alterations in cartilage, synovium, or bone and identify the regulatory nodes most proximal to disease. Animal validation experiments incorporating both sexes, dose–response designs, extended follow-up, tissue-specific measurements, and conditional genetic or pharmacological interventions can test the necessity and sufficiency of candidate pathways in mediating SM effects on OA outcomes. Together, these investigations will distinguish whether SM acts primarily as a risk biomarker, a pathogenic metabolic exposure, or a modifiable therapeutic node. Defining the specific molecular species, target cell types, and causal mechanisms by which SM influences OA susceptibility remains the central translational challenge and the logical next step for this line of inquiry. Such clarification may inform metabolic approaches to OA prevention and intervention.

## 5. Conclusions

Converging evidence from Mendelian randomization, genetic colocalization, and experimental phenotyping supports an association between elevated serum sphingomyelin and OA susceptibility. These findings position SM as a candidate metabolic factor in OA pathogenesis and warrant investigation of lipid-targeted strategies for prevention and intervention.

## Data Availability

The original contributions presented in this study are included in the article/Supplementary Material. Further inquiries can be directed to the corresponding author.

## Supplementary Materials

The following supporting information can be downloaded at https://www.mdpi.com/article/10.3390/biomedicines14102335/s1.
